# Genome analysis of *Campylobacter concisus* strains from patients with inflammatory bowel disease and gastroenteritis provides new insights into pathogenicity

**DOI:** 10.1038/srep38442

**Published:** 2016-12-02

**Authors:** Heung Kit Leslie Chung, Alfred Tay, Sophie Octavia, Jieqiong Chen, Fang Liu, Rena Ma, Ruiting Lan, Stephen M Riordan, Michael C. Grimm, Li Zhang

**Affiliations:** 1 School of Biotechnology and Biomolecular Sciences, University of New South Wales, Sydney, Australia; 2 Helicobacter Research Laboratory, Marshall Centre for Infectious Diseases Research and Training, School of Pathology and Laboratory Medicine, University of Western Australia, Perth, Australia; 3 Gastrointestinal and Liver Unit, Prince of Wales Hospital, University of New South Wales, Sydney, Australia; 4 St George and Sutherland Clinical School, University of New South Wales, Sydney, Australia

## Abstract

*Campylobacter concisus* is an oral bacterium that is associated with inflammatory bowel disease. *C. concisus* has two major genomospecies, which appear to have different enteric pathogenic potential. Currently, no studies have compared the genomes of *C. concisus* strains from different genomospecies. In this study, a comparative genome analysis of 36 *C. concisus* strains was conducted including 27 *C. concisus* strains sequenced in this study and nine publically available *C. concisus* genomes. The *C. concisus* core-genome was defined and genomospecies-specific genes were identified. The *C. concisus* core-genome, housekeeping genes and 23S rRNA gene consistently divided the 36 strains into two genomospecies. Two novel genomic islands, CON_PiiA and CON_PiiB, were identified. CON_PiiA and CON_PiiB islands contained proteins homologous to the type IV secretion system, LepB-like and CagA-like effector proteins. CON_PiiA islands were found in 37.5% of enteric *C. concisus* strains (3/8) isolated from patients with enteric diseases and none of the oral strains (0/27), which was statistically significant. This study reports the findings of *C. concisus* genomospecies-specific genes, novel genomic islands that contain type IV secretion system and putative effector proteins, and other new genomic features. These data provide novel insights into understanding of the pathogenicity of this emerging opportunistic pathogen.

*Campylobacter concisus* is a Gram-negative motile bacterium that grows under both anaerobic and microaerobic conditions with the presence of hydrogen significantly aiding growth^1^. The human oral cavity is the natural colonization site of *C. concisus*, although *C. concisus* may also colonize the intestinal tract in some individuals^2^,^3^.

*C. concisus* has gained increasing attention in recent years due to its association with enteric diseases, in particular inflammatory bowel disease (IBD) which includes Crohn’s disease (CD) and ulcerative colitis (UC). A number of studies reported a significantly higher detection of *C. concisus* by PCR in intestinal biopsies collected from patients with IBD as compared to controls^4^,^5^,^6^,^7^. In addition to IBD, *C. concisus* was frequently isolated from diarrheal stool samples, suggesting its possible role in human diarrheal disease^8^,^9^,^10^,^11^.

Previous studies found that some oral *C. concisus* strains or their toxins were able to damage the intestinal epithelial barrier and induce intestinal epithelial production of proinflammatory cytokines using cell line models^11^,^12^,^13^. These data suggest that translocation of enteric virulent *C. concisus* strains from the human oral cavity to the intestinal tract may cause enteric diseases in some individuals.

Earlier studies found that some *C. concisus* strains had only 42–50% DNA-DNA hybridization value with the reference *C. concisus* strain; however no phenotypic tests were able to differentiate them^14^. These strains were referred to as different genomospecies^15^. *C. concisus* has two genomospecies, which were defined by the analysis of amplified fragment length polymorphisms (AFLP), housekeeping genes and a PCR method targeting the polymorphisms of *C. concisus* 23S rRNA gene^15^,^16^,^17^,^18^,^19^,^20^,^21^,^22^. The two *C. concisus* genomospecies contained both oral and enteric *C. concisus* strains^15^,^16^,^17^,^18^,^19^,^20^,^21^. Strains from the two *C. concisus* genomospecies appear to have different enteric pathogenic potentials. Oral *C. concisus* strains that were invasive to intestinal epithelial cells were found in Genomospecies 2 (GS2)^10^,^11^. GS2 *C. concisus* strains were more often isolated from faecal samples collected from patients with bloody diarrhoea and they were more invasive to intestinal epithelial cells as compared to Genomospecies 1 (GS1) strains^15^,^16^.

Currently, no studies have compared the genomes of *C. concisus* strains from different genomospecies. Identification of *C. concisus* genomospecies-specific genes and other genomic features will provide insights into the evolution and pathogenic potential of this bacterium. We therefore performed comparative genome analysis of 36 *C. concisus* strains including 27 strains that were sequenced in this study and nine publically available *C. concisus* genomes, which revealed new genomic features of *C. concisus* genomospecies and identified novel genomic islands that contain proteins homologous to the type IV secretion system (T4SS) and potential virulence effector proteins.

## Results

### The draft genomes of 27 *C. concisus* strains

The genomes of 27 *C. concisus* strains were sequenced in this study. These 27 *C. concisus* strains were previously isolated in our laboratory from patients with CD, UC and healthy controls and they were randomly selected for inclusion into this study. Ten of these strains were analysed in our previous studies of grouping *C. concisus* strains using housekeeping genes^3^,^6^,^10^,^23^.

The draft genome sizes of these *C. concisus* strains were 1.80 to 2.21 Mb. The contig numbers ranged from 7 to 76. The fold coverage ranged from 83.98 to 230.58. The summaries of the *C. concisus* genomes sequenced in this study are in Table 1.

### The core-genome and accessory genes

The *C. concisus* core-genome was derived from 36 *C. concisus* strains including the 27 *C. concisus* strains sequenced in this study and nine *C. concisus* genomes that are publically available^3^,^6^,^10^,^23^,^24^.

The *C. concisus* core-genome of the 36 *C. concisus* strains consisted of 582 genes, which were 28.7% (582/2025) of the total number of genes present in *C. concisus* strain 13826. The core-genomes of GS1 and GS2 strains had 1,098 and 1,143 genes respectively. The genes in both GS1 and GS2 *C. concisus* core-genomes were evenly distributed amongst different Clusters of Orthologous Groups (Supplementary Fig. S1). The accessory genes in the 36 *C. concisus* strains ranged from 1,163 to 1,521.

### The two *C. concisus* genomospecies identified from analysis of *C. concisus* core-genome, housekeeping genes and 23S rRNA gene

**Figure 1:**
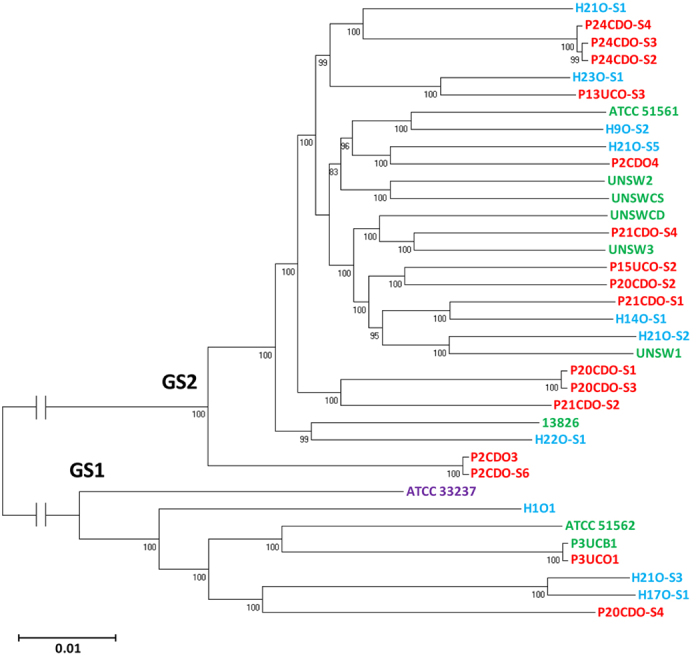
The phylogenetic tree generated based on *C. concisus* core-genome sequences. The phylogenetic tree was generated based on the *C. concisus* core-genome generated from 36 *Campylobacter concisus* strains using Roary^45^. Oral strains from patients with IBD that were sequenced in this study are coloured red. Oral strains from healthy controls that were sequenced in this study are coloured blue. Oral strain ATCC 33237 is coloured purple; this strain was isolated from a patient with gingivitis. Enteric strains are coloured green. The genome of enteric strain P3UCB1, a strain isolated from intestinal biopsies of a patient with UC, was sequenced in this study. The remaining genomes of enteric *C. concisus* strains are publically available. Enteric strain ATCC 51561 was isolated from faecal samples of a healthy individual. Enteric strains UNSW2, UNSW3 and UNSWCD were isolated from patients with CD^24^. The remaining enteric strains were isolated from patients with gastroenteritis. Bootstrap values of more than 70 are indicated on the internal branches. GS1 and GS2 indicate Genomospecies 1 and 2 respectively.

The phylogenetic tree generated based on the core-genome sequences divided the 36 *C. concisus* strains into two genomospecies. Most of the strains belonged to GS2 (77.8%, 28/36) while only eight strains belonged to GS1 (22.2%, 8/36). GS1 and GS2 contained both oral and enteric strains. Some individuals carried *C. concisus* strains from both GS1 and GS2. For example, multiple strains from two individuals (P20CDO-S1, P20CDO-S2, P20CDO-S3, and P20CDO-S4 from a patient with CD as well as H21O-S1, H21O-S2, H21O-S3 and H21O-S5 strains from a healthy individual) were found in different genomospecies (Fig. 1).

**Figure 2:**
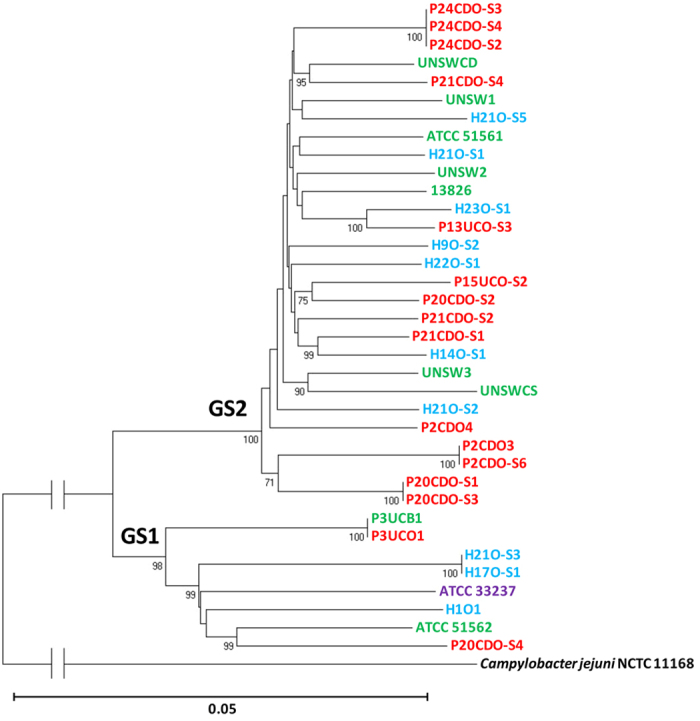
The phylogenetic tree generated based on housekeeping genes of the 36 *Campylobacter concisus* strains. The sequences of six housekeeping genes (*asd, aspA, atpA, glnA, pgi* and *tkt*) were extracted from the 36 *C. concisus* strains and were used to generate the phylogenetic tree using neighbour-joining method, which was performed using molecular evolutionary genetic analysis software version 6.06 (MEGA 6.06) with 1,000 bootstrap replications^47^. Oral strains from patients with IBD that were sequenced in this study are coloured red. Oral strains from healthy controls that were sequenced in this study are coloured blue. Oral strain ATCC 33237 is coloured purple; this strain was isolated from a patient with gingivitis. Enteric strains are coloured green. The genome of enteric strain P3UCB1, a strain isolated from intestinal biopsies of a patient with UC, was sequenced in this study. The remaining genomes of enteric *C. concisus* strains are publically available. Enteric strain ATCC 51561 was isolated from faecal samples of a healthy individual. Enteric strains UNSW2, UNSW3 and UNSWCD were isolated from patients with CD^24^. The remaining enteric strains were isolated from patients with gastroenteritis. Bootstrap values of more than 70 are indicated on the internal branches. *Campylobacter jejuni* strain NCTC11168 was used as an outgroup (GenBank accession no. NC_002163). GS1 and GS2 indicate Genomospecies 1 and 2 respectively.

**Figure 3:**
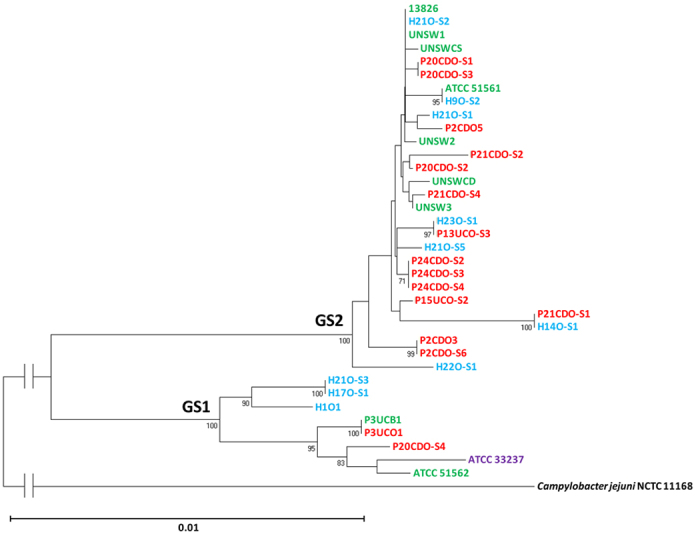
The phylogenetic tree generated based on the sequences of 23S ribosomal RNA genes of the 36 *Campylobacter concisus* strains. The phylogenetic tree was generated based on the sequences of the 23S ribosomal RNA genes. The neighbour-joining method was used to generate the phylogenetic tree, which was performed using Molecular Evolutionary Genetic Analysis software version 6.06 (MEGA 6.06) with 1,000 bootstrap replications^47^. Oral strains from patients with IBD that were sequenced in this study are coloured red. Oral strains from healthy controls that were sequenced in this study are coloured blue. Oral strain ATCC 33237 is coloured purple; this strain was isolated from a patient with gingivitis. Enteric strains are coloured green. The genome of enteric strain P3UCB1, a strain isolated from intestinal biopsies of a patient with UC, was sequenced in this study. The remaining genomes of enteric *C. concisus* strains are publically available. Enteric strain ATCC 51561 was isolated from faecal samples of a healthy individual. Enteric strains UNSW2, UNSW3 and UNSWCD were isolated from patients with CD^24^. The remaining enteric strains were isolated from patients with gastroenteritis. Bootstrap values of more than 70 are indicated on the internal branches. Bootstrap values of more than 70 are indicated on the internal branches. *Campylobacter jejuni* strain NCTC11168 was used as an outgroup (GenBank accession no. NC_002163). GS1 and GS2 indicate Genomospecies 1 and 2 respectively.

Both housekeeping genes and a PCR method targeting the polymorphisms of 23S rRNA gene were previously used to separate *C. concisus* strains into different groups^15^,^16^,^17^,^18^,^19^,^20^,^21^. In this study, we compared the assignment of *C. concisus* strains by housekeeping genes and 23S rRNA gene. The sequences of these housekeeping genes or 23S rRNA gene divided the 36 strains into two clusters, consistent with the GS1 and GS2 grouping assigned based on the *C. concisus* core-genome (Figs 2 and 3).

**Figure 4:**
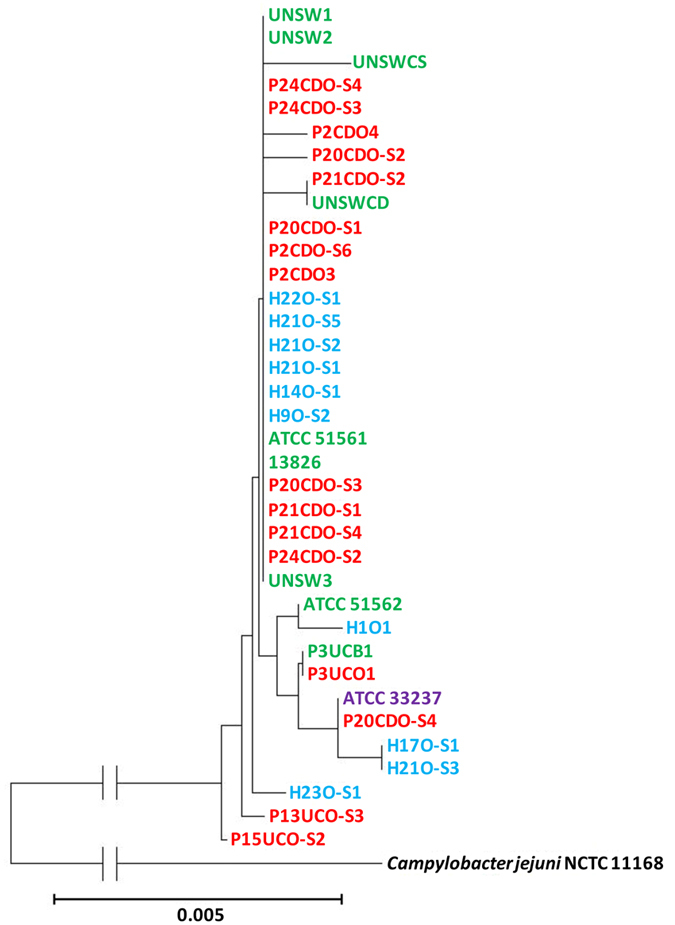
The phylogenetic tree generated based on the sequences of 16S ribosomal RNA genes for the 36 *Campylobacter concisus* strains. The phylogenetic tree was generated based on the sequences of the 16S ribosomal RNA genes. The neighbour-joining method was used to generate the phylogenetic tree, which was performed using Molecular Evolutionary Genetic Analysis software version 6.06 (MEGA 6.06) with 1,000 bootstrap replications^47^. Oral strains from patients with IBD that were sequenced in this study are coloured red. Oral strains from healthy controls that were sequenced in this study are coloured blue. Oral strain ATCC 33237 is coloured purple; this strain was isolated from a patient with gingivitis. Enteric strains are coloured green. The genome of enteric strain P3UCB1, a strain isolated from intestinal biopsies of a patient with UC, was sequenced in this study. The remaining genomes of enteric *C. concisus* strains are publically available. Enteric strain ATCC 51561 was isolated from faecal samples of a healthy individual. Enteric strains UNSW2, UNSW3 and UNSWCD were isolated from patients with CD^24^. The remaining enteric strains were isolated from patients with gastroenteritis. Bootstrap values of more than 70 are indicated on the internal branches. *Campylobacter jejuni* strain NCTC11168 was used as an outgroup (GenBank accession no. NC_002163).

A previous study examining eight *C. concisus* strains found that the 16S rRNA gene was able to differentiate *C. concisus* strains isolated from patients with gastroenteritis and CD^24^. However, in this study, we found that the 16S rRNA gene was unable to differentiate *C. concisus* genomospecies or their related diseases (Fig. 4).

### Genomospecies-specific genes

Using Burrows-Wheeler Aligner, BLASTn and BLASTx, we found that some genes that were present in all GS1 *C. concisus* strains were absent in all GS2 strains and *vice versa*, showing that these were genomospecies-specific genes. The flanking regions of GS1-specific genes were found in the genomes of all GS2 strains on unbroken contigs, and *vice versa*, further confirming that they were truly genomospecies specific.

Of the nine GS1-specific genes, three genes encode phosphate transport proteins (PstS, PstA and PstC). The remaining GS1-specific genes encode hypothetical proteins, transporter proteins and enzymes (Table 2). Fourteen GS2-specific genes were found, including genes that encode a protein involved in regulation of osmolarity (aquaporin Z), a protein involved in pH homeostasis and sodium extrusion (Na^+^/H^+^ antiporter NhaC), twitching motility protein and the others (Table 2).

### CRISPR-associated proteins

Twenty-two *C. concisus* strains, all belonged to GS2, were found to have genes encoding CRISPR-associated proteins. Cas1, Cas2, Cas3 and Cas4a proteins were found in all 22 strains. Cas5h, Csh1 and Csd2/Csh2 proteins were found in most of these 22 strains, Cas6 protein was found in five strains and the remaining seven CRISPR-associated proteins were found in one or two *C. concisus* strains (Table 3).

### Two different genomic islands containing T4SS homologues and putative effector proteins were found in enteric and oral *C. concisus* strains respectively

**Figure 5:**
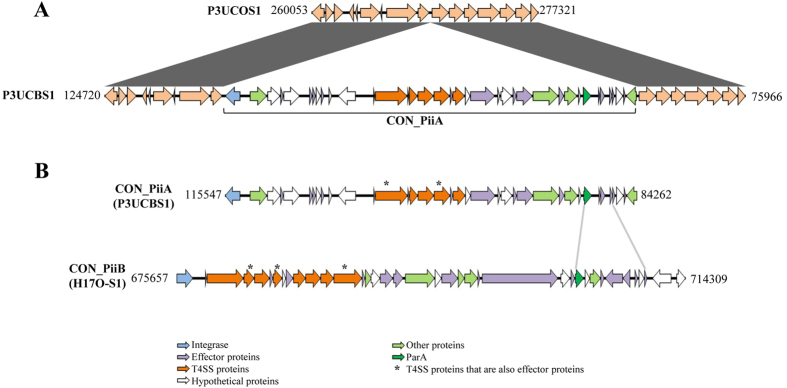
Genomic islands CON_PiiA and CON_PiiB. (**A**) Comparison of proteins in *C. concisus* strains P3UCO1 and P3UCB1 shows the insertion of CON_PiiA island in P3UCB1 strain. The identical proteins in these two strains are shaded in dark grey. (**B**) Proteins in CON_PiiA and CON_PiiB islands. T4SS homologous proteins are coloured orange and the putative effector proteins are coloured purple. The two proteins that had more 40% identities in CON_PiiA and CON_PiiB are shown with light grey lines. The remaining proteins in these two islands had less than 20% amino acid identities.

P3UCO1 and P3UCB1 strains were isolated from saliva and intestinal biopsies of a patient with UC. These two strains were genetically closely related (Fig. 1). Interestingly we found a region in the genome of the enteric strain P3UCB1 that was absent in the genome of the oral strain P3UCO1 (Fig. 5A). The size of this region is 31,286 bp, beginning with an integrase. This region contained five proteins homologous to T4SS proteins from the tumour inducing (Ti) plasmid in plant pathogen *Agrobacterium tumefaciens*, which includes VirB4, VirB8, VirB9, VirB10 and VirB11. Their similarities to the *A. tumefaciens* VirB proteins were 41%, 42%, 29%, 39% and 50% respectively. Furthermore, this region had proteins homologous to the RP4 plasmid conjugative transfer protein TraQ, the plasmid partitioning protein ParA and to various hypothetical proteins. Collectively, these findings showed that this region is a plasmid derived genomic island, which we have named the *C. concisus* plasmid integrative island A (CON_PiiA) (Fig. 5A and Table 4). Two additional enteric *C. concisus* strains, UNSW2 and ATCC 51562 were found to have CON_PiiA based on the annotated proteins. CON_PiiA was identified in 37.5% (3/8) of the enteric *C. concisus* strains isolated from individuals with enteric disease and interestingly none of the oral strains (0/27), which was statistically different (*P* = 0.0086). The core-genomes of multiple oral strains collected from some individuals were genetically similar (Fig. 1), which may lead to biased statistical results. Therefore, we re-analysed the data by considering multiple oral *C. concisus* strains from a given individual as one strain if these strains were in the same small group in Fig. 1. P24CDO-S3, P24CDO-S2 and P24CDO-S4 were considered as one strain, P2CDO3 and P2CDO-S6 were considered as one strain, P20CDO-S1 and P20CDO-S3 were considered as one strain, H21O-S1 and H21O-S5 were considered as one strain. Therefore, the total number of oral strains used for re-analysis was 22 instead of 27. The presence of CON_PiiA in enteric strains isolated from patients with enteric diseases and oral *C. concisus* strains was still significantly different 37.5% (3/8) vs (0/22) (*P* = 0.0138).

We found a second genomic island in oral *C. concisus* strains. A contig in H17O-S1 strain contained the entire island, which was closely examined. Like P3UCB1 strain, H17O-S1 strain had a region containing genes encoding homologues of VirB4 (44% similarity), VirB8 (45%), VirB9 (40%), VirB10 (49%) and VirB11 (49%). Additionally there were proteins homologous to TraQ and various hypothetical proteins. Furthermore, H17O-S1 strain contained genes encoding homologues of VirB5 (33%), VirB6 (32%) and VirD4 (43%) from the Ti plasmid in *A. tumefaciens*, which were not seen in CON_PiiA (Table 4). Repetitive sequences (AGTCCTGGTGAACCCACCA), indicative of attachment sites, were found between an integrase and tRNA-Met-CAT at the positions of 675,445–675,463 bp and 714,647–714,667 bp. Except for two proteins, this region had less than 20% amino acid identities to proteins in CON_PiiA. We named this region *C. concisus* plasmid integrative island B (CON_PiiB), which was 38,653 bp in length (Fig. 5B). The nine VirB proteins and some CON_PiiB proteins were also found in the remaining four oral *C. concisus* strains from two individuals including three strains from one patient with CD (P21CDO-S1, P21CDO-S2, P21CDO-S4), and one strain from a healthy individual (H14O-S1). However, the contigs in the three strains from the patient with CD were not long enough to reveal the entire sequence of CON_PiiB island. CON_PiiB was found in 18.5% (5/27) oral *C. concisus* strains and none of the enteric strains (0/9), which was not statistically significant (*P* > 0.05). The prevalence of CON_PiiB in oral strains isolated from healthy individuals and patients with IBD was 20% (2/10) and 18.8% (3/16) respectively, which was not statistically significant (*P* > 0.05).

Potential effector proteins within CON_PiiA and CON_PiiB islands were found. A number of proteins in both islands had similarities to *Legionella pneumophila* virulence effector proteins, most of which, such as LepB and LepA, are involved in intracellular survival of the pathogen^25^,^26^,^27^,^28^,^29^. One protein had similarities to *Helicobacter pylori* cytotoxin-associated protein A (CagA), which is a virulence factor associated with more severe disease states in *H. pylori* infection^30^. The details of the comparison between proteins in CON_PiiA and CON_PiiB islands and effector proteins are shown in Table 4.

## Discussion

We performed comparative genome analysis of 36 *C. concisus* strains, of which 27 strains were sequenced in this study.

Previous studies using different molecular methods such as AFLP, analysis of housekeeping genes and PCR of the 23S rRNA gene showed that *C. concisus* has two genomospecies^15^,^16^,^17^,^18^,^19^,^20^,^21^. There was some evidence that *C. concisus* strains of these two genomospecies may have different pathogenic potential^15^,^16^,^17^,^18^,^19^,^20^,^21^. For example, strains invasive to intestinal epithelial cells were often found in GS2^10^,^11^. Despite these findings, there is a lack of understanding regarding these two *C. concisus* genomospecies at the genome level.

In this study, for the first time we compared the genomes of *C. concisus* strains from different genomospecies, which revealed new genomic features of this bacterium. We analysed the nine publically available *C. concisus* genomes, together with the genomes of additional 27 *C. concisus* strains that we have sequenced. We generated the *C. concisus* core-genome from these 36 *C. concisus* strains. The core-genome, the sequences of six housekeeping genes and the 23S rRNA gene consistently assigned these *C. concisus* strains into two genomospecies (Figs 1, 2, 3). The enteric strains did not form distinct groups within both genomospecies, further supporting our previous theory that some oral *C. concisus* strains may cause enteric disease when colonizing the intestinal tract^3^,^31^,^32^. The previous study examining eight *C. concisus* strains reported that 16S rRNA gene of *C. concisus* strains was able to differentiate *C. concisus* strains isolated from patients with CD and gastroenteritis, this was not observed in our study where 36 *C. concisus* strains were examined (Fig. 4)^24^.

We found nine genes that were specific to GS1 *C. concisus* strains and fourteen genes that were specific to GS2 *C. concisus* strains, some of which encode proteins that may contribute to the survival and pathogenicity of *C. concisus* (Table 2). For example, three of the nine GS1-specific genes encode proteins involved in phosphate transport (PstS, PstA, PstC), suggesting that strains of GS1 and GS2 may differ in their phosphate uptake. Aquaporin Z was found in all GS2 *C. concisus* strains, but not in any GS1 strains. Aquaporin Z is a protein that moves water across bacterial membranes to maintain intracellular osmotic pressure^33^. The finding that GS2 *C. concisus* strains have aquaporin Z suggests that they may have enhanced abilities in adapting to environments where osmolarity frequently changes.

The type I CRISPR system, which has the Cas3 protein, was found in 78.6% (22/28) of GS2 *C. concisus* strains (Table 3). However, the number of CRISPR-associated proteins between *C. concisus* strains varied. Cas6, an endoribonuclease that generates RNAs for defense in the type I CRISPR system, was present in only five *C. concisus* strains. CRISPR system provides acquired immunity to plasmids and phages^34^,^35^. The CRISPR proteins found in *C. concisus* strains do not seem to be related to CON_phi2 prophage that contains the zonula occludens toxin gene^31^. The *C. concisus* Zot was found to damage intestinal epithelial barrier and affect the function of macrophages and the *zot* gene was detected in *C. concisus* strains from both GS1 and GS2^11^,^23^,^36^.

Two novel *C. concisus* genomic islands were identified in this study. CON_PiiA and CON_PiiB islands were found in both GS1 and GS2 *C. concisus* strains. CON_PiiA was found in 37.5% (3/8) of enteric strains isolated from patients with enteric diseases including two patients with IBD and one patient with gastroenteritis, but not in the 27 oral *C. concisus* strains, a difference that was statistically significant. CON_PiiA was not found in ATCC 51561, an enteric strain isolated from faecal samples of a healthy individual. CON_PiiB was found in 18.5% (5/27) of oral *C. concisus* strains and none of the enteric strains, this difference did not reach statistical significance. Collectively, these data suggest that the CON_PiiA island may preferably integrate into enteric *C. concisus* strains isolated from patients with enteric diseases. However, the numbers of enteric *C. concisus* strains included in this study were small, larger numbers of enteric *C. concisus* strains need to be examined to confirm this finding.

Both CON_PiiA and CON_PiiB islands contained T4SS homologous proteins. The T4SS system is used by microorganisms to transport macromolecules such as proteins or DNA across the cell envelope^37^. T4SS may be involved in plasmid conjugation, uptake or release of DNA or transfer effector proteins into host cells^38^. The well-studied *H. pylori cag* pathogenicity island encodes proteins homologous to VirB2, VirB4, VirB5, VirB7, VirB9, VirB10, VirB11 and VirD4; these proteins deliver effector proteins such as CagA to host cells through the formation of a pilus^39^. Putative effector proteins similar to *L. pneumophila* and *H. pylori* virulence effector proteins were found in both CON_PiiA and CON_PiiB islands. The virulence effector proteins in *L. pneumophila* are mainly involved in bacterial survival within macrophages^25^,^26^,^27^,^28^,^29^. *H. pylori* CagA virulence factor is associated with gastric cancer^30^. Given that the two novel *C. concisus* genomic islands found in this study contained proteins similar to T4SS and their effector proteins found in human pathogens, CON_PiiA and CON_PiiB islands are likely to be involved in *C. concisus* virulence. However, the putative effector proteins found in CON_PiiA and CON_PiiB islands had similarities to only a fragment of CagA and *L. pneumophila* effector proteins. Their true virulence requires confirmation by characterization of individual proteins in these islands.

To our knowledge, this is the first study examining the genomes of *C. concisus* strains of different genomospecies. We sequenced the genomes of 27 *C. concisus* strains and performed comparative genome analysis of 36 *C. concisus* strains. We generated the core-genome from 36 *C. concisus* strains. The *C. concisus* core-genome, six housekeeping genes and 23S rRNA gene consistently divided the 36 strains into two genomospecies. We also identified GS1 and GS2 *C. concisus* specific genes. Furthermore, we identified two novel genomic islands that contained T4SS homologous proteins and putative effector virulence proteins; CON_PiiA appeared to be associated with enteric *C. concisus* strains isolated from patients with enteric diseases. The new *C. concisus* genomic features obtained from this study provide novel insights into understanding of the pathogenicity of this emerging opportunistic pathogen.

## Methods

### *C. concisus* strains used for genome sequencing

*C. concisus* strains sequenced in this study were isolated from saliva samples or intestinal biopsies in our previous studies^3^,^6^,^11^,^22^. The genomes of 27 *C. concisus* strains were sequenced. *C. concisus* strains were grown on Horse Blood Agar (HBA) plates as previously described^1^. DNA was extracted from each *C. concisus* strain using the Gentra Puregene Yeast/Bacteria Kit according to the manufacturer’s instructions (Qiagen, Hilden, Germany). The quality of DNA was checked using Nanodrop and Qubit Fluorometer. Bacterial genomic DNA (1 ng) was used for genomic library generation in accordance with the Nextera XT protocol (Ver. May 2012). Libraries were sequenced for a 250 bp paired-end sequencing run using Nextera XT V2 on the MiSeq Personal Sequencer running version 1.1.1 MiSeq Control Software (Illumina Inc., San Diego, CA, USA). Reagent contamination was controlled by barcoding all DNA samples and preparation of barcoding index primers for a single use. The quality of reads was assessed based on the Phred quality score of the reads. The reads mapping fold coverage was calculated using qualimap_v2.0^40^. We aimed to get a fold coverage of at least 50X for each genome, which was shown to be adequate for characterization of genomes^41^.

### Draft genome assembly and identification of *C. concisus* pan- and core-genome

In addition to the above 27 *C. concisus* strains sequenced in this study, nine *C. concisus* genomes that are available in NCBI database were also included for analysis, of which seven genomes were from a previous study^24^. The accession numbers of these nine *C. concisus* genomes are ANNF00000000, ANNJ00000000, ANNE00000000, AENQ00000000, ANNG00000000, ANNH00000000, ANNI00000000, CP000792.1, NZ_CP012541.1. The genomes of strains 13826 and ATCC 33237 (accession numbers CP000792.1, NZ_CP012541.1) were fully sequenced and the remaining genomes were draft genomes. Thus, a total of 36 *C. concisus* strains were analysed in this study including 27 oral strains and nine enteric strains.

The raw reads were assembled using St. Petersburg genome assembler to obtain the draft genomes (SPAdes, Ver. 3.6.1)^42^ (Table 1). Gene annotation was performed using a combination of Rapid Annotations using Subsystems Technology server (RAST, Ver. 2.0) and Prokka (Ver. 1.11)^43^,^44^. The pan- and core-genome for the 36 *C. concisus* strains were defined by the Rapid large-scale prokaryote pan-genome analysis software (Roary, Ver. 3.5.7)^45^. The genome function analysis was performed as described previously^46^. Briefly, the protein sequences were extracted from the annotated genomes and blasted against the NCBI COG database (ver. 2014). Genes with COG assignment were then categorised in a list of functional groups.

### Phylogenetic analysis based on the *C. concisus* core-genome, sequences of housekeeping genes, 23S and 16S rRNA genes

The phylogenetic tree based on the *C. concisus* core-genome was generated using Roary^45^. The neighbour-joining method was used to generate phylogenetic trees based on housekeeping genes, 23S rRNA genes and 16S rRNA genes of the 36 *C. concisus* strains examined in this study, which were performed using Molecular Evolutionary Genetic Analysis software version 6.06 (MEGA 6.06) with 1,000 bootstrap replications^47^. The six housekeeping genes were previously shown to be able to define *C. concisus* genomospecies, including aspartase A (*aspA*), glutamine synthetase (*glnA*), transketolase (*tkt*), aspartate semialdehyde dehydrogenase (*asd*), ATP synthase F1 alpha subunit (*atpA*) and glucose-6-isomerase (*pgi*)^18^. The sequences of housekeeping genes, 23S and 16S rRNA genes from a *Campylobacter jejuni* strain (GenBank accession no. NC_002163) were used as an outgroup.

### Identification of genomospecies-specific genes

The annotated genes of the 36 *C. concisus* strains representing the two genomospecies were compared using Roary to determine candidate genes that were specific to GS1 or GS2. A GS1-specific gene refers to a gene that is present in all GS1 strains and absent in all GS2 strains analysed in this study. Similarly, a GS2-specific gene refers to a gene that is present in all GS2 strains and absent in all GS1 strains. To confirm the presence and absence of genomospecies-specific genes, the assemblies from each of the genome were searched with BLASTn (BLAST+, Ver. 2.2.31) and BLASTx (BLAST+, Ver. 2.2.31)^48^. To ensure the absence of genomospecies-specific genes were not due to issues with assemblies and sequencing artefacts, raw reads were mapped with Burrows-Wheeler Aligner (BWA, Ver. 0.7.12)^49^. Finally flanking regions of the absent genes were confirmed to be located on the same contig.

### Identification of genomic islands and the putative effector proteins

Two *C. concisus* genomic islands containing T4SS homologous proteins were identified in this study, which were based on the comparison of the flanked genes in *C. concisus* strains, the presence of integrases and attachment sites, the sizes of the regions, and the presence of plasmid-associated genes. Clustal Omega was used to compare protein sequences between islands^50^. The effector proteins were identified by comparing the proteins in the identified genomic islands with the proteins in the T4SS secretion system effector protein database SecReT4 using WU-BLAST on default settings^51^.

### Statistical analysis

Fisher’s exact test (two tailed) was used to compare the prevalence of CON_PiiA and CON_PiiB islands in enteric and oral *C. concisus* strains. Statistical analysis was performed using GraphPad Prism 6 software (San Diego, CA).

### GenBank sequence submission

Raw reads of the 27 *C. concisus* strains sequenced in this study were submitted to Sequence Reads Archive in GenBank under the BioProject number PRJNA348396.

## Additional Information

**How to cite this article**: Chung, H. K. L. *et al*. Genome analysis of *Campylobacter concisus* strains from patients with inflammatory bowel disease and gastroenteritis provides new insights into pathogenicity. *Sci. Rep.* **6**, 38442; doi: 10.1038/srep38442 (2016).

**Publisher's note:** Springer Nature remains neutral with regard to jurisdictional claims in published maps and institutional affiliations.

## Supplementary Material

Supplementary Information

## Figures and Tables

**Table 1:** Summary of the 27 *C. concisus* genomes sequenced in this study.

Strain ID	Health status	No. of Contigs	N50	Genome size (Mb)	Fold coverage
H1O1*	Healthy	21	225,994	1.86	144.81
H9O-S2*	Healthy	32	172,447	2.03	169.43
H14O-S1*	Healthy	33	322,175	1.98	117.90
H17O-S1*	Healthy	15	986,534	1.95	129.68
H21O-S1	Healthy	38	112,460	2.04	109.13
H21O-S2	Healthy	40	138,316	2.03	133.83
H21O-S3	Healthy	25	369,626	1.98	160.43
H21O-S5	Healthy	73	75,277	2.12	115.50
H22O-S1	Healthy	76	162,003	2.14	202.49
H23O-S1	Healthy	33	298,329	2.14	166.47
P2CDO3*	CD	72	277,481	2	155.83
P2CDO4*	CD	30	223,561	2.09	230.58
P2CDO-S6	CD	53	278,451	2.01	189.64
P3UCB1*	UC	17	361,314	1.82	118.62
P3UCO1*	UC	7	569,893	1.8	102.09
P13UCO-S3*	UC	57	214,312	2.06	183.54
P15UCO-S2*	UC	27	451,303	1.96	142.96
P20CDO-S1	CD	75	227,524	2.08	204.22
P20CDO-S2	CD	55	266,446	2.05	155.16
P20CDO-S3	CD	71	197,586	2.21	194.63
P20CDO-S4	CD	10	1,298,069	1.92	90.93
P21CDO-S1	CD	32	215,002	2.02	223.50
P21CDO-S2	CD	68	332,372	2.15	171.43
P21CDO-S4	CD	38	129,907	1.93	83.98
P24CDO-S2	CD	26	184,230	1.95	186.95
P24CDO-S3	CD	57	85,614	1.99	167.75
P24CDO-S4	CD	40	136,251	1.94	129.65

Draft genomes were assembled using St. Petersburg genome assembler (SPAdes, Ver. 3.6.1). Letters P and H in strain ID indicate strains isolated from patients with inflammatory bowel disease and healthy controls respectively. O indicates oral strains isolated from saliva samples and B indicates a strain isolated from intestinal biopsies. These strains were isolated from our previous studies^3^,^6^.

^*^Indicates strains used in a previous study using housekeeping genes to group *C. concisus* strains^18^. CD: Crohn’s disease. UC: Ulcerative colitis.

**Table 2:** Genomospecies-specific genes.

GS1-specific gene products	Locus tag
Transporter, AbgT family	CCON33237_0883
Hypothetical protein	CCON33237_0734
Hypothetical protein	CCON33237_1772
Tellurite-resistance/dicarboxylate transporter, TDT family	CCON33237_1254
Transcriptional regulator, Crp family	CCON33237_1253
Putative NADH dehydrogenase	CCON33237_1252
Phosphate ABC transporter, permease protein PstA	CCON33237_1171
Phosphate ABC transporter, permease protein PstC	CCON33237_1170
Phosphate ABC transporter, periplasmic substrate-binding protein PstS	CCON33237_1169
**GS2-specific gene products**	**Locus tag**
LemA protein	CCC13826_1702
Twitching motility protein	CCC13826_1584
Hydroxylamine reductase	CCC13826_1540
Aspartate racemase	CCC13826_1511
DNA-3-methyladenine glycosylase 1	CCC13826_0272
Oxidoreductase, FAD/FMN-binding	CCC13826_0436
Aquaporin Z	CCC13826_1636
Glyoxalase II	CCC13826_1402
Beta-lactamase HcpA (Cysteine-rich 28 kDa protein)	CCC13826_2180
Rhomboid family protein	CCC13826_1263
Beta-aspartyl peptidase	CCC13826_0178
Na+/H+ antiporter NhaC	CCC13826_0177
Periplasmic protein	CCC13826_0895
PAS/PAC sensor signal transduction histidine kinase	CCC13826_0721

GS: genomospecies. Locus tag: GS1-specific genes locus tag refers to the locus in *C. concisus* strain ATCC 33237; GS2-specific genes locus tag refers to the locus in *C. concisus* strain 13826.

**Table 3:** CRISPR-associated proteins in *Campylobacter concisus* strains.

**CRISPR**															
Strain ID	Cas1	Cas2	Cas3	Cas4a	Cas5h	Cas6	Csh1 family	Csh2 family	Csd2/Csh2 family	Csm1 family	Csm2 family	Csm3	Csm4 family	Csm5 family	TM1812
P2CDO5	+	+	+	+	+		+		+						
P13UCO-S3	+	+	+	+	+		+		+						
P15UCO-S2	+	+	+	+	+		+		+						
P20CDO-S1	+	+	+	+	+				+						
P20CDO-S2	+	+	+	+	+		+		+						
P20CDO-S3	+	+	+	+	+				+						
P21CDO-S1	+	+	+	+	+	+	+		+						+
P21CDO-S2	+	+	+	+	+		+		+						
P24CDO-S2	+	+	+	+	+		+		+						
P24CDO-S3	+	+	+	+	+		+		+						
P24CDO-S4	+	+	+	+	+		+		+						
H9O-S2	+	+	+	+					+						
H14O-S1	+	+	+	+	+		+		+						
H21O-S1	+	+	+	+	+	+	+		+						+
H21O-S2	+	+	+	+	+		+		+						
H21O-S5	+	+	+	+	+	+	+		+	+	+	+	+	+	
H23O-S1	+	+	+	+	+		+		+						
13826	+	+	+	+	+		+	+							
ATCC51561	+	+	+	+					+						
UNSW1	+	+	+	+	+		+		+						
UNSW2	+	+	+	+					+						
UNSWCS	+	+	+	+		+			+						

All *C. concisus* strains that have CRISPR-associated proteins belonged to Genomospecies 2. Letters P and H in strain ID indicate oral strains isolated from patients with inflammatory bowel disease and healthy controls respectively. The remaining five strains were enteric strains isolated from patients with Crohn’s disease and gastroenteritis. A positive sign (+) indicates the presence of a gene.

**Table 4:** Putative effector proteins and other proteins in CON_PiiA and CON_PiiB genomic islands.

CON_PiiB (strain H17O-S1)	Island protein size (AA)	Effector	Effector size (AA)	E-value	Homology to known bacterial effector#	Bacterial strain
Integrase	384	AnkI/legAS4	545	0.027	49% (101AA) (408–508)	*Legionella pneumophila*
Massive surface protein MspG	433	LepB	1294	8.50E-09	44% (406AA) (590–995)	*L. pneumophila*
Hypothetical protein@	328	LepB	1294	0.0018	42% (250AA) (883–1132)	*L. pneumophila*
TraQ@	78					
Hypothetical protein	412	LaiA/SdeA	1545	0.056	44% (299AA) (1059–1357)	*L. pneumophila*
Hypothetical protein	79	Ceg4	364	0.097	63% (27AA) (19–45)	*L. longbeachae*
Hypothetical protein	79					
Hypothetical protein	124					
Hypothetical protein	79	TPR family protein	532	0.00093	51% (61AA) (472–532)	*Coxiella burnetii*
Hypothetical protein	83					
Hypothetical protein	446	LepB	1294	0.0017	48% (304AA) (637–940)	*L. pneumophila*
Hypothetical protein	38					
VirB4	821	LepB	1294	8.70E-05	46% (364AA) (859–1222)	*L. pneumophila*
Hypothetical protein	40					
VirB8	216					
VirB9	407					
VirB10@	407					
Hypothetical protein	68	YlfA/legC7	425	0.035	72% (32AA) (337–368)	*L. pneumophila*
VirB11	315					
Hypothetical protein	136	YlfB/legC2	405	0.023	53% (91AA) (152–242)	*L. pneumophila*
Hypothetical protein	621					
Hypothetical protein	65	Lem19	416	0.034	54% (26AA) (162–187)	*L. pneumophila*
Hypothetical protein	285	hypothetical	230	0.049	46% (162AA) (48–209)	*L. pneumophila*
Hypothetical protein	91					
Hypothetical protein	406					
DNA topoisomerase I	651	CagA	1230	0.0016	39% (234AA) (655–878)	*Helicobacter pylori* G27
Single-stranded DNA-binding protein	133					
EcoRI methylase/methyltransferase	332					
Hypothetical protein	67	Ceg2	274	0.026	52% (33AA) (107–139)	*L. pneumophila*
ParA*	214	PieA/lirC	699	0.1	44% (188AA) (425–612)	*L. pneumophila*
Hypothetical protein	45					
Hypothetical protein	119					
Hypothetical protein	78					
Hypothetical protein*	67	hypothetical	205	0.00029	62% (29AA) (19–47)	*L. pneumophila*
Hypothetical protein	188	hypothetical	216	0.079	49% (51AA) (123–173)	*L. pneumophila*
Hypothetical protein	53					
Initiator replication protein	267					
CON_PiiB (strain H17O-S1)	Island protein size (AA)	Effector	Effector size (AA)	E-value	Homology to effector#	Bacterial strain
Integrase	417					
Hypothetical protein	38					
VirB4	929					
VirB5@	264	LepA	1119	0.015	45% (100AA) (235–334)	*L. pneumophila*
VirB6	388					
Hypothetical protein	81	hypothetical	162	0.0018	54% (50AA) (18–67)	*L. pneumophila*
VirB8	234	Ceg28	1159	0.089	43% (136AA) (90–225)	*L. pneumophila*
Hypothetical protein	91					
Hypothetical protein	182	AnkD/legA15	473	0.0045	45% (176AA) (295–470)	*L. pneumophila*
VirB9@	315					
VirB10	381					
VirB11	333					
VirD4	717	LaiA/SdeA	1506	0.00056	53% (91AA) (1131–1221)	*L. pneumophila*
Hypothetical protein	73					
Cag pathogenicity island protein 12@	156					
TraQ@	234					
Hypothetical protein	323	LepB	1294	6.60E-07	46% (273AA) (840–1112)	*L. pneumophila*
Hypothetical protein	248	hypothetical	311	0.1	49% (88AA) (177–264)	*L. pneumophila*
DNA topoisomerase III	748					
Hypothetical protein	170					
Hypothetical protein	415	CagA	1230	5.60E-05	50% (229AA) (649–877)	*Helicobacter pylori* G27
Hpa2 protein@	166					
DNA primase	334					
Hypothetical protein	60	MavC	482	0.023	69% (29AA) (138–166)	*L. pneumophila*
Helicase	1922	LepB	1294	0.0096	58% (101AA) (906–1006)	*L. pneumophila*
Hypothetical protein	236					
Hypothetical protein	99	Lem27	564	0.048	48% (62AA) (322–383)	*L. pneumophila*
ParA*	220					
Hypothetical protein	128					
Abieii	271					
Hypothetical protein	89	hypothetical	494	0.068	55% (47AA) (445–491)	*L. pneumophila*
TraR	446	RavB	296	0.016	47% (118AA) (77–194)	*L. pneumophila*
Hypothetical protein	187	SdhB	1875	0.014	47% (145AA) (1035–1179)	*L. pneumophila*
Hypothetical protein	73	hypothetical	208	0.00014	53% (60AA) (10–69)	*L. pneumophila*
Hypothetical protein	143					
Hypothetical protein*	67	hypothetical	205	0.00029	46% (39AA) (19–47)	*L. pneumophila*
Hypothetical protein	478					
Hypothetical protein	232					

AA: amino acid.

^#^The homology of putative effector proteins in CON_PiiA and CON_PiiB islands to known bacterial effector proteins based on BLASTp was expressed as % similarity (the number of amino acids used for comparison) (the start and end position of the known bacterial effector proteins that matched).

^@^Proteins predicted to contain a signal peptide.

^*^The two proteins in CON_PiiA and CON_PiiB had more than 40% identities and the remaining proteins in these two islands had less than 20% identities.
