# Erratum: Genome analysis of *Campylobacter concisus* strains from patients with inflammatory bowel disease and gastroenteritis provides new insights into pathogenicity

**DOI:** 10.1038/srep41113

**Published:** 2017-02-16

**Authors:** Heung Kit Leslie Chung, Alfred Tay, Sophie Octavia, Jieqiong Chen, Fang Liu, Rena Ma, Ruiting Lan, Stephen M. Riordan, Michael C. Grimm, Li Zhang

Scientific Reports 6: Article number: 38442; 10.1038/srep38442 published online: 12 02 2016; updated: 02 16 2017.

This Article contains an error in Table 4. The text in the first row ‘CON_PiiA (strain P3UCB1)’ was incorrectly given as ‘CON_PiiB (strain H17O-S1).’

