# Supplementary material for: Genome analysis of Campylobacter concisus strains from patients with inflammatory bowel disease and gastroenteritis provides new insights into pathogenicity
Source: Sci Rep. 2016 Dec 2;6:38442. doi: 10.1038/srep38442 (PMC5133609; doi:10.1038/srep38442)
Supplement: Supplementary Information [file srep38442-s1.pdf]

Genome analysis of *Campylobacter concisus* strains from patients with inflammatory bowel disease and gastroenteritis provides new insights into pathogenicity

Heung Kit Leslie Chung[1#], Alfred Tay[2#,] Sophie Octavia[1], Jieqiong Chen[1], Fang Liu[1], Rena Ma[1], Ruiting Lan[1], Stephen M Riordan[3], Michael C Grimm[4], Li Zhang[1]*

1. School of Biotechnology and Biomolecular Sciences, University of New South Wales, Sydney, Australia

2. Helicobacter Research Laboratory, Marshall Centre for Infectious Diseases Research and Training, School of Pathology and Laboratory Medicine, University of Western Australia, Perth, Australia

3. Gastrointestinal and Liver Unit, Prince of Wales Hospital, University of New South Wales, Sydney, Australia

4. St George and Sutherland Clinical School, University of New South Wales, Sydney, Australia

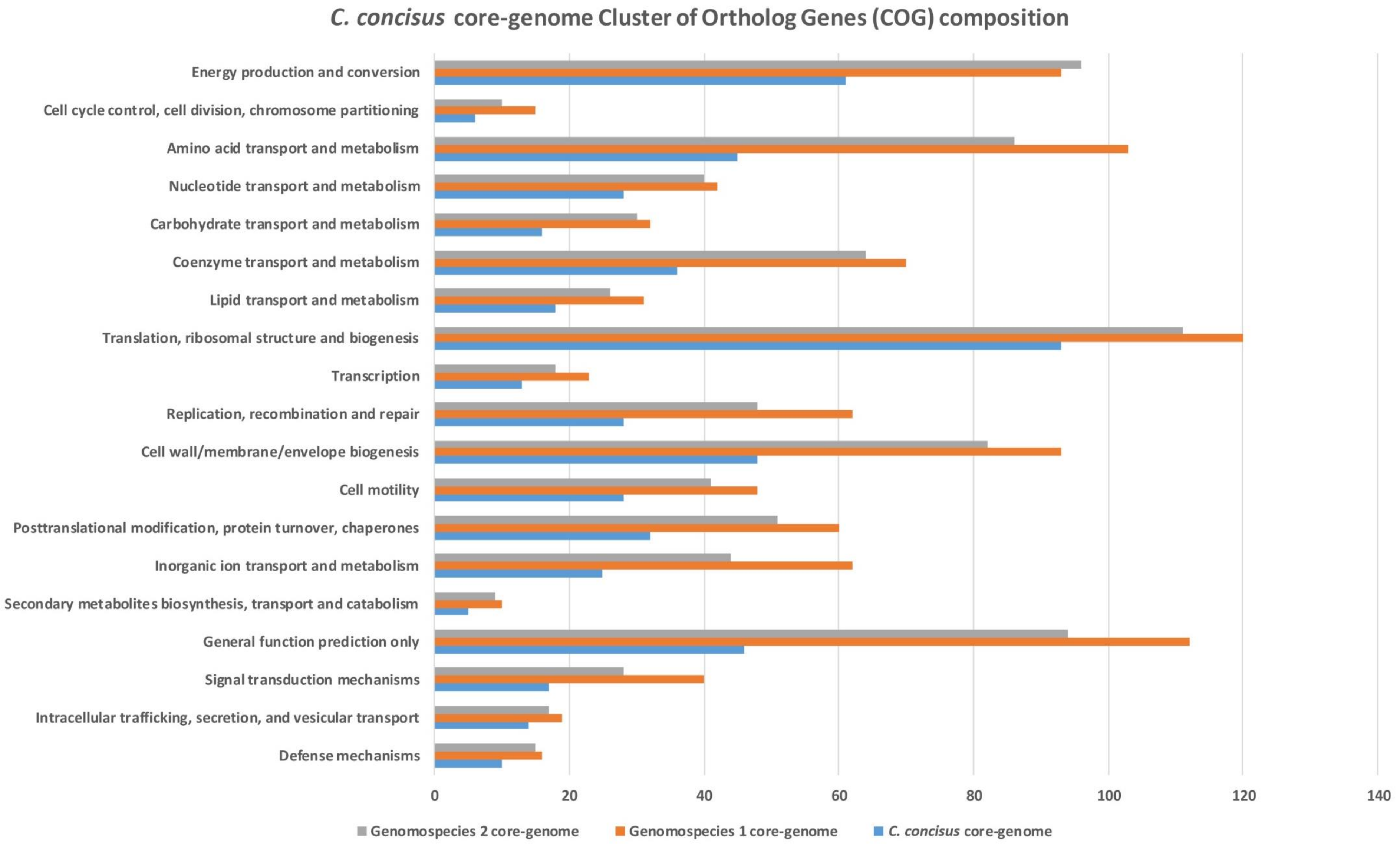

Supplementary Figure S1. The distribution of *C. concisus* core-genome genes in different functional categories.

The numbers represent number of genes in each Cluster of Ortholog Genes. The grey bar represents Genomospecies 2, orange bar represents Genomospecies 1 and the blue bar represents all *C. concisus* strains examined in this study.
